# Inflammation and Vascular Calcification Causing Effects of Oxidized HDL are Attenuated by Adiponectin in Human Vascular Smooth Muscle Cells

**DOI:** 10.22088/IJMCM.BUMS.8.1.39

**Published:** 2019-06-28

**Authors:** Noor Hanisa Harun, Gabriele Ruth Anisah Froemming, Hapizah Md Nawawi, Suhaila Abd Muid

**Affiliations:** 1 Faculty of Medicine, Universiti Teknologi MARA, Cawangan Selangor, Sungai Buloh Campus, Selangor, Malaysia.; 2 Faculty of Medicine and Health Sciences, Universiti Malaysia Sarawak, Kota Samarahan, Sarawak, Malaysia.; 3 Institute of Pathology, Laboratory and Forensic Medicine (I-PPerForM), Universiti Teknologi MARA, Sungai Buloh Campus, Selangor, Malaysia.

**Keywords:** HAoVSMCs, oxidized high-density lipoprotein, osteoblastic transdifferentiation, vascular calcification, adiponectin

## Abstract

The role of oxidized high- density lipoprotein (oxHDL) and the protective effects of adiponectin in terms of vascular calcification is not well-established. This study was conducted to investigate the effects of oxHDL with regard to inflammation and vascular calcification and to determine the protective role of adiponectin in attenuating the detrimental effects of oxHDL. Cell viability, mineralization, and calcification assays were conducted to optimize the concentration of oxHDL. Then, human vascular smooth muscle cells (HAoVSMCs) were incubated with β-glycerophosphate, HDL, oxHDL, adiponectin, or the combination of oxHDL with adiponectin for 24 h. Protein expression of IL-6, TNF-α, osterix, RUNX2, ALP, type 1 collagen, osteopontin, osteocalcin, WNT-5a, NF-ĸβ(p65), cAMP and STAT-3 were measured by ELISA kits. OxHDL induced vascular calcification by promoting the formation of mineralization nodules and calcium deposits in HAoVSMCs. This was accompanied by an increased secretion of IL-6, osterix, WNT-5a and NF-ĸβ (p65). Interestingly, these detrimental effects of oxHDL were suppressed by adiponectin. Besides, incubation of adiponectin alone on HAoVSMCs showed a reduction of inflammatory cytokines, osteoblastic markers (RUNX2, osterix and osteopontin), WNT-5a and NF-ĸβ (p65). This study exhibits the ability of oxHDL in inducing inflammation and vascular calcification and these detrimental effects of oxHDL can be attenuated by adiponectin.

Vascular calcification, the deposition of calcium in the intima layer of arteries, accelerates the progression of atherosclerotic plaque formation and has been considered as the most important event occurring during the advanced atherosclerosis stage (1). This plaque formation will lead to the formation of thrombotic occlusion which will narrow the blood vessel. The plaque would become unstable and rupture may occur; the blood flow to the brain or heart can be blocked; causing stroke or an infarct (2). Vascular calcification is a major risk factor for coronary artery disease and a major cause of death in patients with chronic kidney failure (3). It is believed to be initiated by transdifferentiation of vascular smooth muscle cells (VSMCs) to osteoblast-like cells (4) followed by calcium-hydroxyapatite crystal deposition in the medial layer of arteries. VSMCs, the predominant resident cells, play an important role in maintaining the blood flow and regulating contraction and relaxation. However, it has been shown that VSMCs isolated from atherosclerotic regions lost their physiological characteristics and so leading to stiffness of the arteries and reduced blood flow (5). These isolated cells started to behave like osteoblast cells by secreting bone-related protein biomarkers including transcription factors for osteoblast differentiation and maturation (4-6βα^7^,^ 8^).

HDL exerts many beneficial effects against the progression of cardiovascular disease by inhibiting the initial step of atherosclerosis progression through preventing accumulation of cholesterol in the macrophages via its reverse cholesterol efflux activity (8). While in the late stages of atherosclerosis, HDL plays its role in the prevention of vascular calcification by inhibiting the transdifferentiation of VSMCs (6). Furthermore, HDL also could reduce the alkaline phosphatase (ALP) secretion and signal transducer and activator of transcription 3 (STAT-3) activity in calcifying VSMCs isolated from atherosclerotic mice (7). However, HDL is prone to modification especially oxidation, which might change its protective properties into pro-atherogenic ones. The presence of oxidized high-density lipoprotein (oxHDL) in atherosclerotic plaques has been suspected as one of the major factors that enhanced the progression of atherosclerosis (1), even though little actual reports have been published. However, there is a study reporting that the presence of oxHDL heightened the activity of ALP and calcium deposition in already calcifying murine vascular cells (1). Furthermore, increased HDL concentration in coronary artery disease patients by drugs such as fibrates and niacin failed to demonstrate its beneficial effects in reducing the risk of cardiovascular events. It is suggested that even though the quantity of HDL increases, the molecular structure of HDL is disturbed causing it to lose its beneficial components and become malfunctioning (9).

Adiponectin, an anti-inflammatory and anti-atherogenic adipokine, has been known to exert many beneficial effects against the progression of atherosclerosis and its level in the circulation is inversely correlated with the progression of cardiovascular disease (10). Adiponectin reduced calcium deposition induced by TNF- and inorganic phosphate which significantly repressed the progression of vascular calcification (11). However, the protective effects of adiponectin on vascular calcification induced by oxHDL *in vitro* are still unclear. Therefore, the objectives of this study were to investigate the ability of oxHDL in inducing calcification in human vascular smooth muscle cells (HAoVSMCs), to study the effectiveness of adiponectin in attenuating the detrimental effect of oxHDL by assessing the protein expression of inflammatory biomarkers such as IL-6 and TNF- and osteogenic protein biomarkers such as ALP, osteopontin, type 1 collagen and osteocalcin in HAoVSMCs and to determine the possible pathways involved in oxHDL detrimental effects by measuring the protein expression of Runt-related transcription factor 2 (RUNX2), osterix and WNT-5a.

## Materials and methods

**Cell Culture**

Primary adult human aortic vascular smooth muscle cells, HAoVSMCs (Lot no: 400Z012.2; Promocell, USA) was used in this present study. The cells were grown and cultured in Smooth Muscle Cell Growth Medium (Promocell, USA) supplemented with 1 % antibiotics antimycotics solution (Sigma-Aldrich, USA) according to the protocol. The cells were maintained in 5% CO2 incubator with 37 °C humidified chamber, until passage 6. Accutase (Innovative Cell Technologies, USA) was used to detach the cells during subculturing. Upon stimulation with treatment groups, the culture medium was changed to Dulbecco's Modified Eagle Medium, DMEM (Gibco, ThermoFisher Scientific; USA) containing 15 % foetal bovine serum, FBS (Sigma-Aldrich, USA) and 1 % antibiotic antimycotic solution.

**Oxidation of HDL**

Firstly, commercially obtained HDL (Merck, Germany), certified to be from healthy donors, was dialyzed in the dark for 24 h with three buffer exchanges to remove any preservative agents. Then HDL (1 mg/ml protein) was incubated with 50 M copper sulphate (Sigma-Aldrich, USA) for 4 h at 37 °C in the dark. About 2.5 mM of EDTA (Sigma-Aldrich, USA) was added to stop the oxidation before the HDL mixture was dialyzed against phosphate buffered saline (0.01 M phosphate buffer, 0.0027 M potassium chloride and 0.137 M sodium chloride) (Sigma-Aldrich, USA) for 24 h in the dark with 3 buffer exchanges. Then, the oxHDL was stored at 4 °C and used within a week. The degree of oxidation was measured by OxiSelect^™^ TBARS Assay Kit (Cell Biolab Inc, USA). The average value of TBARs in oxHDL for this experiment was 119.9+21 nmol/l/mg protein of malondialdehyde.

**Cell viability assay (MTS assay)**

The cytotoxic effects of oxHDL and adiponectin on HAoVSMCs were measured using MTS assay kit (CellTiter 96® AQueous One Solution Reagent; Promega, USA) by measuring optical density (OD) at 490 nm using a Perkin Elmer Victor X5 2030 Multilabel Luminescence Microplate Reader.

**Mineralization assay**

About 3.5 x 104 HAoVSMCs cells/well were transduced into osteoblast-like cells through incubation with osteogenic media or oxHDL for 14 days. The positive control was composed of DMEM with 15 % FBS, 1 % antibiotic, 10 mM µ-glycerophosphate and 0.1 mM ascorbic acid (Sigma-Aldrich, USA). The other wells were contained DMEM with 15 % FBS, 1 % antibiotic and different concentrations of oxHDL (10, 25, 50 and 100 µg/ml protein).

The mineralization assay was performed according to previous study (12). Briefly, the transduced cells were fixed with 4 % formaldehyde (in PBS) at 4 C for 45 min. The fixative was removed, and the cells were washed with distilled water 3 times. Then, the cells were stained with 1 ml of 2 % alizarin red S, pH 4.1- 4.3 (Merck, Germany) at room temperature for 20 min with gentle shaking. Then, the dye was removed, and the cells were washed twice with distilled water. The images of the stained cells were captured before the dye in each well was extracted by using the acetic acid method. The concentration of the extracted alizarin red staining was measured by comparing the absorption readings of the samples with the readings of a standard curve prepared by serial diluting of alizarin red staining and measured at 450 nm wavelength.

**Calcification assay**

Deposited calcium content in the stimulated cells was measured by Calcium Colorimetric Assay Kit (Sigma-Aldrich, USA) which is based on the o-cresolphthalein complexone method as previously described by Yang et al. (13). After the stimulation with oxHDL (10, 25, 50 and 100 μg/ml protein oxHDL) for 14 days, the cells were decalcified with 0.6 N hydrochloric acid (HCl) for 24 h. The calcium ions content in the HCl supernatant was measured by using the Calcium Colorimetric assay mentioned above. Then, the cells were washed with PBS three times and were solubilized with 0.1 N sodium hydroxide (NaOH). The protein contents were measured using the BCA protein assay kit (Pierce, USA). The calcium content was normalized to the total protein content (2).

**Quantification of ALP, osteopontin, osteocalcin, type 1 collagen, RUNX2, osterix, WNT-5a, IL-6, TNF-α, NF-ĸβ p65, cAMP, STAT-3 by using ELISA**

HAoVSMCs were cultured in 6 well culture plates until 90 % confluency was reached. Then, the cells were incubated for 24 h with 10 mM β-glycerophosphate (Merck, Germany), HDL (100 μg/ml protein), oxHDL (100 μg/ml protein), adiponectin (5-15 μg/ml) alone and the combination of oxHDL (100 μg/ml protein) and adiponectin. Human recombinant trimeric adiponectin was purchased from Abcam, UK. Cells incubated with β-glycerophosphate and culture medium served as positive and negative control, respectively. The targeted proteins were measured by ELISA according to the manufacturer’s instruction. All ELISA kits were purchased from Cloud-Clone, USA; except for the osteocalcin and STAT-3 ELISA kits, which were purchased from Invitrogen^TM^ eBioscience, UK; and NF-ĸβ (p65) ELISA kit was purchased from Cayman Chemical, USA. The supernatant of each sample was collected to measure IL-6, TNF-α, type 1 collagen, ALP, osteocalcin, osteopontin and WNT-5a. As for osterix, STAT-3, cAMP and RUNX2, cytosolic protein samples were used and extracted using AllPrep® RNA/Protein kit (Qiagen, Germany). While for the transcription factor, NF-ĸβ (p65), nuclear extracts were used and extracted by nuclear extraction kit (Cayman Chemical, USA).

**Statistical analysis**

Data were analysed using SPSS version 21.0. ANOVA test was performed to assess overall differences between the different groups of treatment followed by Bonferroni Post-HOC test. Significant value was set at P <0.05.

## Results

**Non-toxic concentrations of oxHDL and adiponectin**

**Fig. 1:**
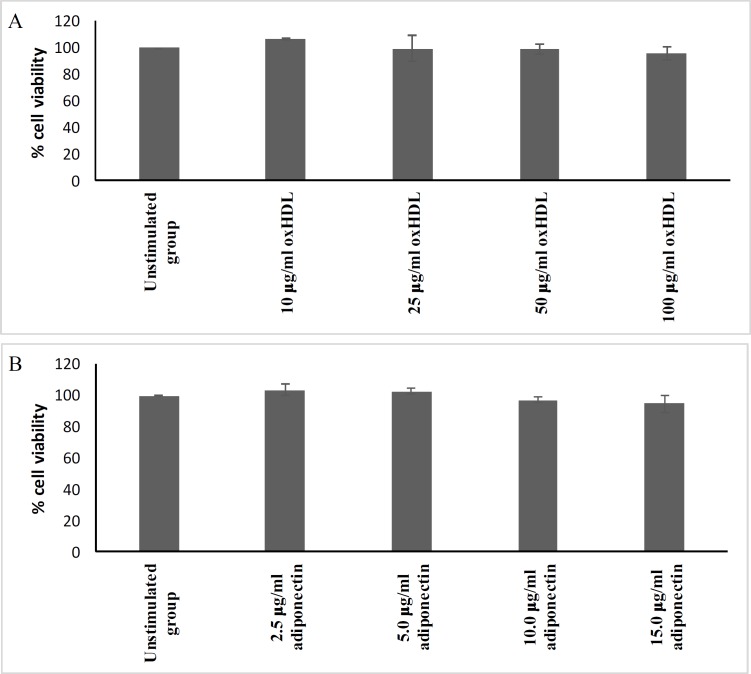
**Cytotoxicity of oxHDL and adiponectin on HAoVSMCs.** HAoVSMCs were incubated with various concentrations of (A) oxHDL and (B) adiponectin for 24 h. Then, the percentage of viable cells was determined by CellTiter 96® AQueous One Solution, measured at OD 490 nm. The results are shown as mean ± SEM of 3 experiments with triplicate determination

The MTS test was performed to determine the non-toxic concentration range of oxHDL and adiponectin for further analysis. Figure 1 shows the range of concentrations used for both oxHDL (A) and adiponectin (B). OxHDL at 10-100 μg/ml protein exhibited a cell viability above 95 %. Adiponectin concentrations ranging from 2.5–15 μg/ml maintained a cell viability above 95 %. Therefore, this concentration range for oxHDL was used for mineralization and calcification experiments. While the range of adiponectin was from 5–15 μg/ml and these concentrations were used for the treatment of cells for protein expression of inflammation and osteogenic biomarkers.

**Copper sulphate (Cu** ^2+^ **) oxidized HDL induces the formation of mineralization nodules and incorporation of calcium into HAOVSMCs**

**Fig. 2:**
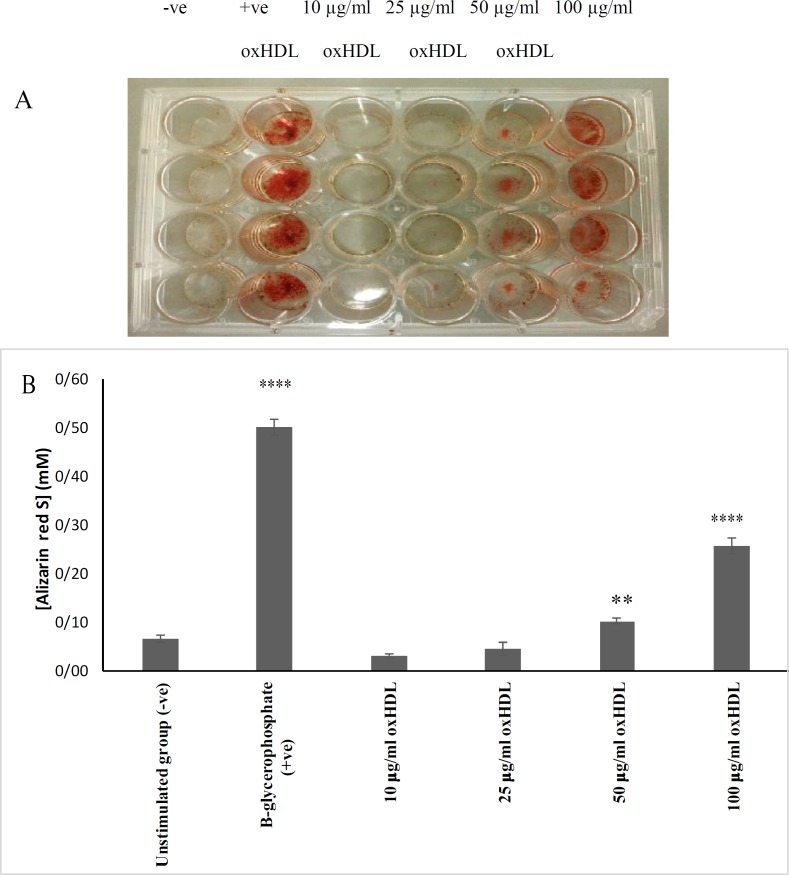
**Effects of oxHDL on mineralization of HAoVSMCs.** HAoVSMCs were incubated with culture medium (negative controls), β-glycerophosphate (positive controls) or different concentrations of oxHDL (10, 25, 50, 100 μg/ml protein) for 14 days. The medium was changed for every 3 days. A: HAoVSMCs were stained with 2 % alizarin red for detection of mineral nodules indicated by the presence of red colour. B: alizarin red dye that stained the monolayer of the cells was extracted by using acetic acid method and the results are shown as mean   SEM of 3 experiments with triplicate readings. *P ≤ 0.05, **P ≤ 0.01, ***P ≤ 0.001, and ****P ≤ 0.0001 in comparison with unstimulated group. -ve indicates negative control, and +ve indicates positive control

Observation of mineral nodule formation by VSMCs and other cells residing in the arteries has been used as an early indicator of the occurrence of vascular calcification before further downstream investigation been carried out. This study showed that HAoVSMCs formed mineralized nodules when incubated with 25, 50 and 100 μg/ml protein of oxHDL (Figure 2A). Visual inspection showed the formation of red-stained alizarin red S/calcium complexes in cells incubated with the positive control and 50 and 100 μg/ml protein of oxHDL (Figure 2A), whereby 100 μg/ml protein of oxHDL exhibited the highest intensity of alizarin red S staining which was significantly higher in comparison with the negative control (P <0.0001) but less intense than the positive control, β-glycerophosphate (P <0.0001) (Figure 2B). β-glycerophosphate is a well- known inducer in generating *in vitr*o models of calcifying VSMCs. The addition of β-glycerophosphate generate high phosphorus environment in the medium, which is important for hydroxyapatite crystal formation. This phosphorous also induces the ALP activity, core binding factor α 1 (*Cbfα1*) gene expression, mineral nodules formation and calcium deposition in VSMCs, which mimic *in vitro* mineralization by osteoblasts (14).

**Fig. 3:**
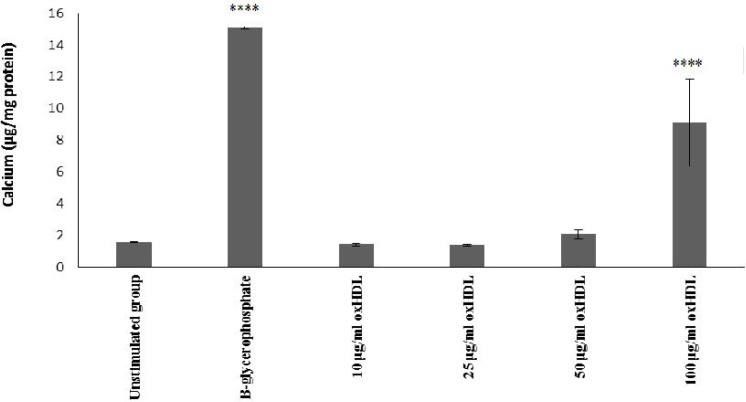
**Effects of oxHDL on calcium incoporation in HAoVSMCs**. HAoVSMCs were incubated with culture medium (un-stimulated, negative control), β-glycerophosphate (positive control) or different concentrations of oxHDL (10, 25, 50, 100 μg/ml) for 14 days. Calcium deposition was analyzed by using calcium colorimetric assay and the results are shown as mean   SEM of 3 experiments with triplicate determination. *P ≤ 0.05, **P ≤ 0.01, ***P ≤ 0.001, and ****P ≤ 0.0001 in comparison with un-stimulated group

In addition, incubation with 100 μg/ml protein oxHDL showed a significant calcium deposition in comparison with unstimulated group (P <0.0001) which was concurrent with the mineralization assay (Figure 3). Therefore, the concentration of 100 μg/ml oxHDL was selected for further studies measuring the protein expression of pro-inflammatory and osteogenic biomarkers. 

**Effects of oxHDL and adiponectin on the secretion of the pro-inflammatory cytokines IL-6 and TNF-a**

**Fig. 4:**
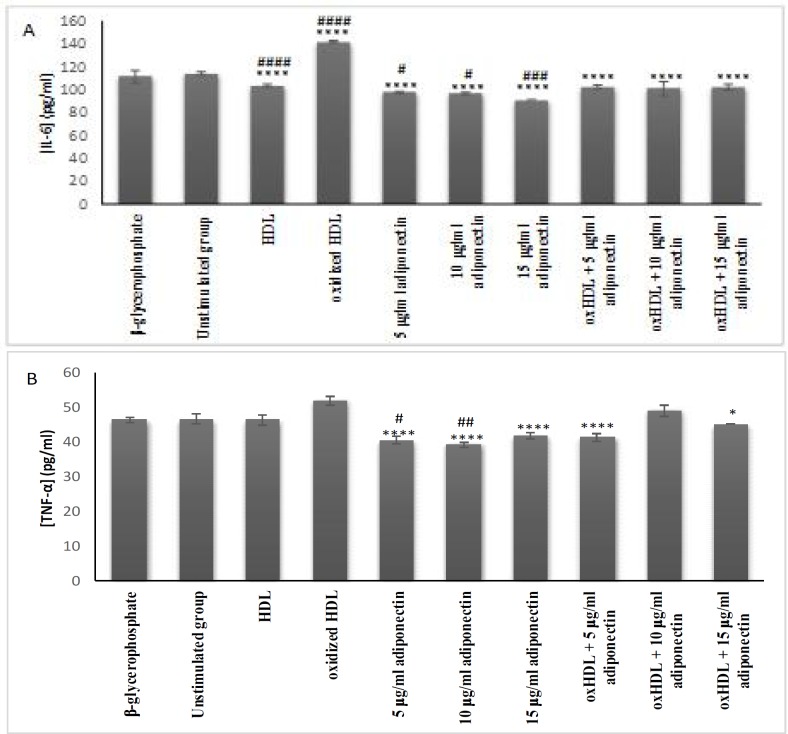
**Effects of oxHDL and adiponectin on the secretion of pro-inflammatory cytokines.** A: IL-6; B: TNF-α. About 3x10^5^ cells/well were treated for 24 h with β-glycerophosphate (positive controls), culture medium (un-stimulated, negative control), HDL (100 μg/ml), oxHDL (100 μg/ml), adiponectin (5, 10 and 15 μg/ml), and combination of oxHDL (100 μg/ml) with adiponectin (5, 10 and 15 μg/ml). The cell supernatant was collected and used for soluble protein expression by ELISA Data are expressed as mean  SEM of 3 experiments. *P ≤ 0.05, **P ≤ 0.01, ***P ≤ 0.001, ****P≤0.0001. *indicate the comparison with oxHDL group, ^#^indicate the comparison with un-stimulated group (media+cells)

Elevation of pro- inflammatory cytokine secretion is known as one of the factors that heightens the progression of atherosclerosis. In this study, oxHDL significantly increased the secretion of IL-6 when compared with HDL (P <0.0001) and unstimulated cells (P <0.0001) (Figure 4A). Adiponectin exhibited a lower IL-6 protein expression in comparison with the oxHDL (P <0.0001). Besides, co-incubation of oxHDL with 5, 10 and 15 µg/ml of adiponectin interestingly resulted in lower secretion of IL-6 in comparison with oxHDL alone (P <0.0001). It shows that adiponectin could suppress the effect of oxHDL on IL-6 secretion.

In contrast to the effect on IL-6 expression, oxHDL showed a non- significant increase of TNF- α secretion in comparison with the negative controls. Therefore, the potential reduction activity of adiponectin towards oxHDL could not be proven even though co-incubation of oxHDL with 5 (P <0.0001) and 15 (P <0.05) μg/ml adiponectin showed significant lower TNF-α values than the oxHDL treated cells. Besides, incubation of cells with adiponectin (5-15 μg/ml) alone also exhibited even lower TNF-α protein expression in comparison with oxHDL (P <0.0001). However, only the lower concentration of adiponectin (5 and 10 μg/ml) showed significant lower secretion when compared with unstimulated group (P <0.05 and P <0.01, respectively).

**Fig. 5:**
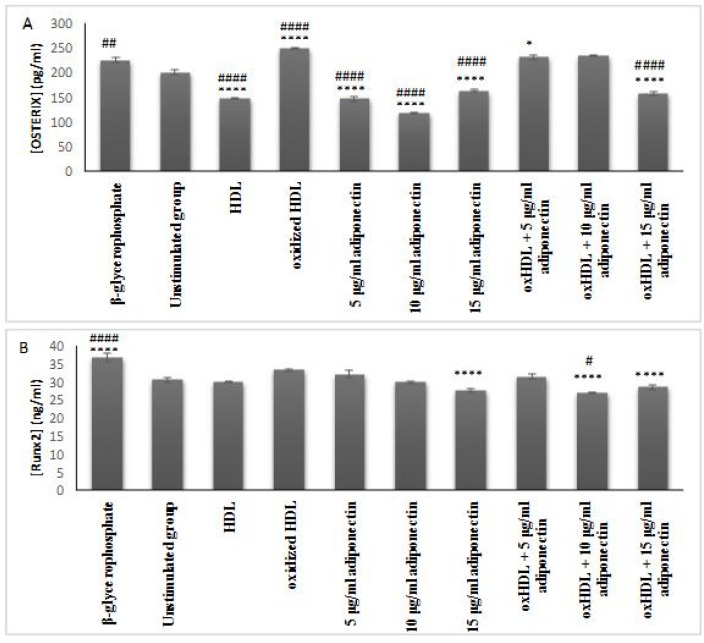
Effects of oxHDL and adiponectin on the secretion of osteoblast transcription factor (A) Osterix and (B) Runx2. About 3x105 cells/well were treated for 24 h with β-glycerophosphate (positive control), culture media (unstimulated, negative control), 100 μg/ml protein HDL, 100 μg/ml protein oxHDL, 5, 10 and 15 μg/ml adiponectin, and combination between 100 μg/ml oxHDL with 5, 10 and 15 μg/ml adiponectin. The cell supernatant was collected and used for protein detection by specific ELISA kits. The results shown in mean   SEM of 3 experiments. *p≤0.05, **p≤0.01, ***p≤0.001, ****p≤0.0001. *indicates the comparison with oxHDL group, #indicates the comparison with un-stimulated group

**oxHDL induced the secretion of osterix rather than RUNX2 in HAoVSMCs, while adiponection suppressed both**

RUNX2 and osterix are the most potent transcription factors for osteoblast differentiation and maturation, respectively. Previous studies have spotted the presence of RUNX2 (15) and osterix (16) in transdiffrentiated VSMCs indicating the cells have been transformed into osteoblast like cells. However, in this study, oxHDL significantly increased the secretion of osterix (P<0.0001 (Figure 5A), while for RUNX2, it only showed a higher trend of expression in comparison with both cells incubated with culture medium and HDL (Figure 5B). It shows that oxHDL favoured to initiate the transcription of osterix and this effect was reduced by the presence of high concentration of adiponectin (15 μg/ml) (P<0.0001). Besides, incubation with adiponectin alone (5-15 μg/ml adiponectin) also showed a very much lower secretion of osterix in comparison with the control group (P<0.0001). Similar results were observed in the cells incubated with HDL (P<0.0001). 

**Fig. 6:**
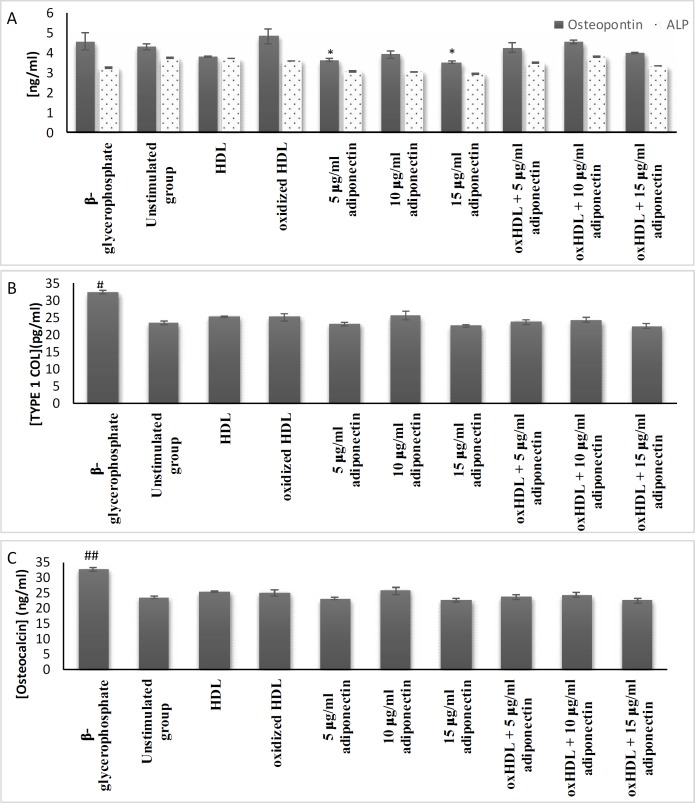
**Effects of oxHDL and adiponectin on the secretion of bone associated protein**. A: ALP and osteopontin; B: type 1 collagen; C: osteocalcin. About 3x10^5^ cells/well were treated for 24 h with β-glycerophosphate (positive control), media (un-stimulated, negative control), 100 μg/ml HDL, 100 μg/ml oxHDL, 5, 10 and 15 μg/ml adiponectin, and combination of 100 μg/ml oxHDL with 5, 10 and 15 μg/ml adiponectin. The cytosolic protein was extracted using AllPrep® RNA/Protein kit (Qiagen) and was quantified by using specific ELISA kit. The results are shown as mean SEM of 3 experiments. *P ≤ 0.05, **P ≤ 0.01, ***P ≤ 0.001, and **** P≤ 0.0001. *indicates the comparison with oxHDL group, ^#^indicates the comparison with unstimulated group

**Fig. 7:**
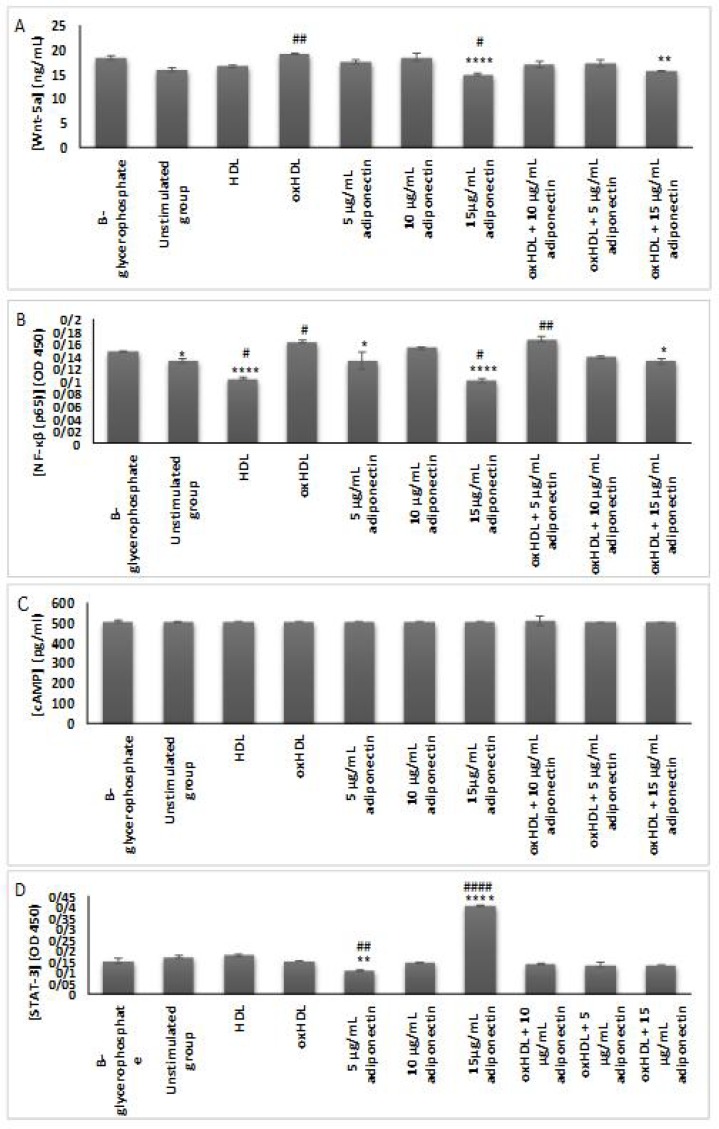
**Effects of oxHDL and adiponectin on the secretion of different proteins.** A: WNT-5a; B: NF-ĸβ (p65); C: cAMP; D: STAT-3. About 3x10^5^ cells/well were treated for 24 h with β-glycerophosphate (positive control), media (un-stimulated, negative control), 100 μg/ml HDL, 100 μg/ml oxHDL, 5, 10 and 15 μg/ml adiponectin, and combination of 100 μg/ml oxHDL with 5, 10 and 15 μg/ml adiponectin. The targeted protein of WNT-5a, cAMP, STAT-3 and NF-ĸβ (p65) were quantified by using specific ELISA kit. The results are shown as mean SEM of 3 experiments. *P ≤ 0.05, **P ≤ 0.01, ***P ≤ 0.001, and ****P≤ 0.0001. *indicates the comparison with oxHDL group, ^#^indicates the comparison with un-stimulated group

This showed that adiponectin and HDL could have protective properties against the production of osterix in vascular cells, while oxHDL could be the antagonist by promoting the secretion of osterix. 

For the RUNX2 assay, only the co-incubation of oxHDL and 10 μg/ml adiponectin showed a significant lower secretion in comparison with the unstimulated group (P <0.05). However, the protective role of adiponectin in preventing RUNX2 secretion cannot be proven because, firstly oxHDL did not increase the secretion of RUNX2, and secondly the incubation of adiponectin alone on the cells did not show any significant reduction of RUNX2. Therefore, oxHDL and adiponectin may not be involved in regulating RUNX2 in this type of cell.

**Effects of oxHDL and adiponectin on the secretion of bone associated proteins**

Osteopontin, ALP, type 1 collagen, and osteocalcin are bone-associated proteins that are commonly expressed by osteoblasts and osteocytes. These proteins are also important in anchoring calcium to the matrix during bone mineralisation process. Based on Figure 6, the presence of oxHDL is not affecting the expression of all the bone associated proteins targeted in this study. Only the osteopontin protein expression showed a slightly higher trend than cultured medium alone. Adiponectin alone had lower osteopontin protein expression than oxHDL. Overall, all the targeted boneassociated proteins investigated in this study were not affected by the induction of oxHDL and adiponectin.

**Effects of oxHDL and adiponectin on the secretion of WNT-5a, cAMP, STAT-3 and NF-ĸβ (p65)**

Evaluation on the secretion of WNT-5a, cAMP, STAT-3 and NF-ĸβ (p65) were performed to elucidate the possible pathways involved in the regulation of inflammation and vascular calcification. Results obtained showed that oxHDL upregulated the secretion of WNT-5a (P <0.01) and NF-ĸβ (p65) (P <0.05), and these effects were reduced in the presence of high concentration of adiponectin (15 μg/ml) (P<0.01 and P <0.05, respectively) (Figure 7 A and B). Incubation of HAoVSMCs with high adiponectin concentration (alone) showed lower secretion of these proteins which indicates that adiponectin has protective effects on the cells, whether in the normal environment or during induction with oxHDL (P <0.05 and P <0.0001; respectively). While treatment with HDL showed lower secretion of NF-ĸβ (p65) in comparison with unstimulated (P <0.05) and oxHDL group (P <0.001). However, there were no changes or differences of cAMP secretion between all treatment groups. This indicates that cAMP might not be involved in the regulation of vascular calcification in this study. In terms of STAT-3 secretion, lower concentration of adiponectin (5 μg/ml) showed a significant reduction (compare to control P <0.01), however higher concentration of adiponectin (15 μg/ml) significantly increased the STAT-3 secretion (P<0.001)+.

## Discussion

Oxidation of HDL is one of the modifications that reverses the normal physiological function of HDL in preventing cardiovascular disease including atherosclerosis. Data obtained from this study showed that a high concentration of oxHDL (100 μg/ml) induces the formation of mineral nodules and elevates calcium deposition of HAoVSMCs which mimics the biological activity of bone cells. In bone, incorporation of calcium inside osteoblast cells activates intracellular calcium signal which induces the nucleation of minerals and formation of hydroxyapatite at matrix vesicles which will later be released out of the cells for normal bone regulation (17). Under normal physiological conditions, VSMCs secrete matrix vesicles, however, these matrix vesicles do not mineralize due to the presence of calcification inhibitors (matrix GLA protein and fetuin-A) which prevent any formation of mineralized nodules on the surface of the cells (18). Therefore, it is suggested that the formation of hydroxyapatite mineral nodules, which is the main form of minerals found in vascular cells in this study is the pathological indicator for vascular calcification 

The secretion of mineralized nodules was due to transdifferentiation of HAoVSMCs to osteoblast-like cells. The present results showed that 100 μg/ml protein of oxHDL enhanced the activation of osterix, an important transcription factor for osteoblast maturation. This may be due to the elevated secretion of the pro-inflammatory cytokine IL-6 which promotes the secretion of WNT-5a and then activates the secretion of osterix and NF-ĸβ (p65) as indicated in this present study. IL-6 has been shown to be actively involved in inducing vascular calcification and enhanced osteogenic differentiation of vascular-related cells such as human umbilical artery smooth muscle cell (19). Therefore, oxHDL promotes vascular calcification by inducing an inflammatory state in HAoVSMCs through the secretion of IL-6. Besides,the study done by Parhami et al. (2002) showed the ability of HDL to partially inhibit the IL-6-induces activation of signal transduction involved in accelerating the progression of vascular calcification. This might explain why the group incubated with HDL showed slightly lower IL-6 secretion in comparison with unstimulated group. It is also suggested that inflammation induced by cytokines and the occurrence of vascular calcification act in a vicious cycle, which means vascular calcification also can promote the secretion of IL-6 (20).

Inflammation, caused by oxHDL, in this study elevated the secretion of WNT-5a in HAoVSMCs. IL-6 was shown to initiate the secretion of WNT-5a in melanoma cells and human bone marrow stromal cells, respectively (21, 22). High secretion of WNT-5a is one of the indicators of the severity of atherosclerotic progression as it was found to be higher in advanced atherosclerotic plaque regions (23). It was also reported that the serum of atherosclerotic patients usually contains higher WNT-5a protein than normal individuals (23). Interestingly, WNT-5a is also involved in inducing vascular calcification of smooth muscle cells through the non-canonical WNT-5a/ROR2 signaling pathway which supports our findings (23, 24). Furthermore, oxLDL which is a very well-known protagonist in atherosclerosis progression, also upregulates the mRNA expression of *WNT-5a* in THP-1 cells and monocyte-derived macrophages (23). Perhaps, oxHDL exhibited similar action as oxLDL in term of *WNT-5a* induction. Furthermore, WNT-5a is an important ligand that is involved in the activation of osteoblast cell differentiation (25). On the other hand, WNT-5a could also activate the secretion of inflammatory cytokines such as IL-1β, IL-6, IL-8, and macrophage inflammatory protein-1β (MIP-1β) through its non-canonical Wnt signaling (26) which intensifies the progression of vascular calcification. In the non-canonical pathway, WNT-5a is postulated to release Ca^2+^ by activating phospholipase C. The elevation of Ca^2+^ in the cells activates either calmodulin-dependent protein kinase II (CAMKII) or protein kinase C (PKC) which might play a pathogenic role in atherosclerosis (23). Besides, the binding of WNT-5a also could activate NF-κB signaling pathway as found in inflamed vascular endothelial cells (27). NF-κB normally exists in the cytosol in the form of homo- and heterodimeric complexes of the Rel family proteins, including p50, p52, p65/RelA, c-Rel, and RelB. Normally, NF-κB is in a heterodimer structure composed of p50 and p65/RelA proteins in which the p65/RelA subunit has transactivation activity (28). This molecule is in an inactive form due to the attachment of the inhibitor of kappa B proteins (Iĸβs). However, inflammation causes this inhibitor to be phosphorylated and consequently detach from the NF-ĸβ structure, allowing NF-ĸβ to penetrate the nucleus and activate the transcription of certain genes (29). The present study showed that treatment of oxHDL elevated the amount of activated NF-ĸβ. This might be due to the increased secretion of WNT-5a and IL-6 in the environment. Little is known on the effects of oxHDL in inducing the secretion of NF-ĸβ (p65) and its connection with the activation of osterix. However, other studies showed that induction of ox-LDL promoted the translocation of NF-ĸβ (p65) into the nucleus which was accompanied with elevation of osterix gene expression (30). Perhaps oxHDL has an action similar to oxLDL in terms of NF-ĸβ (p65) and osterix, which requires further analysis. Besides, HDL has been proven to have protective effects in reducing the translocation of activated NF-ĸβ (p65) and osterix in the present study. This is supported by the finding from Robbesyn et al. where HDL decreased the translocation of NF-ĸβ (p65) and degradation of Iĸβ induced by oxidized LDL in rabbit smooth muscle cells (31).

Both RUNX2 and osterix are critical transcription factors for bone formation by osteoblast cells (17). Increased secretion of these proteins has been detected in calcifying cells residing in the atherosclerotic lesion, thus indicating transforming of smooth muscle cells into osteoblast-like cells (5). The role of inflammatory cytokines in activating Wnt signaling which upregulates the expression of both RUNX2 and osterix has been suggested previously (3). However, in the present study, oxHDL only enhanced the secretion of osterix while showing a neutral effect on RUNX2 secretion. These current findings are equivalent with the results obtained by Taylor et al. where oxLDL co-incubated with β-glycerophosphate also only showed up-regulation of osterix without giving any effect towards *RUNX2* expression (32). Perhaps, oxidized lipoprotein only regulates osterix expression without inducing *RUNX2* in HAoVSMCs transdifferentiation. OxHDL also exhibited a neutral effect on all targeted osteoblast-related proteins: osteopontin, osteocalcin, type 1 collagen, and ALP. This may be due to the fact that RUNX2 is not affected by the treatment with oxHDL at least in the present study. In bone cells (osteoblast), RUNX2 is a potent transcription factor in promoting the secretion of bone-related proteins targeted in this study (5).

**Fig. 8:**
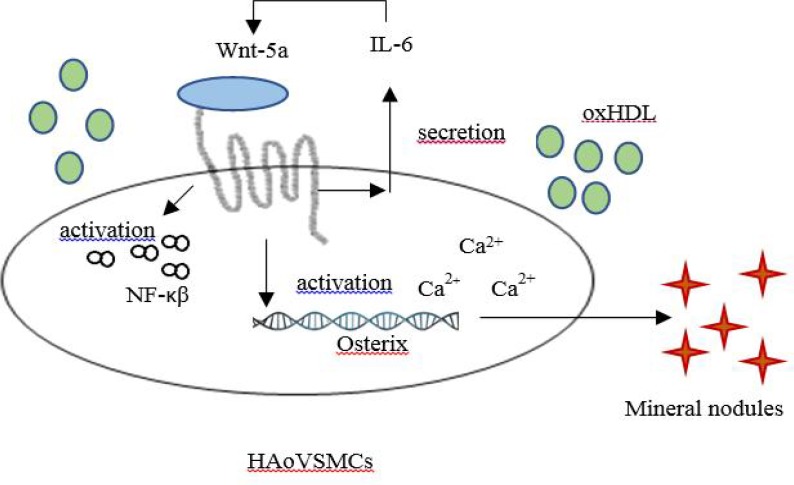
Possible pathway of oxHDL in inducing vascular calcification

Overall, oxidation causes HDL to lose its protective effects and starts to promote pro-atherosclerotic properties in terms of vascular calcification. In this study, oxHDL promoted mineralization and calcification of HAoVSMCs by elevating IL-6, WNT-5a, NF-ĸβ (p65), and osterix secretion as illustrated in Figure 8. However, the detrimental effects of oxHDL in this experiment was suppressed by the presence of trimeric adiponectin. It shows that co-incubation of oxHDL with adiponectin suppressed the secretion of IL-6, osterix, NF-ĸβ (p65), and WNT-5a. Similar results were obtained during the incubation of adiponectin alone in comparison with the un-stimulated group. High secretion of WNT-5a is usually observed in obese individuals where it prevents differentiation of pre-adipocytes (adipogenesis) leading to type 2 diabetes (33). *In vitro* studies showed that induction of high concentration of adiponectin in 3T3-L1 cells reduced the secretion of WNT-5a (34). Besides, the correlation between adiponectin and Wnt-5a could be clearly observed in adiponectin-transgenic mice, where these mice express lower Wnt-5a, while the endogenous adiponectin is highly expressed in comparison with the wild-type mice (34). Perhaps, WNT-5a secretion induced by oxHDL in the current study is also reduced due to the presence of trimeric adiponectin. Exogenous induction of adiponectin into *ApoE*-/-mice showed a significant suppression of *NF-κB* (p65) activation, followed by decreased gene and protein expression of pro-inflammatory markers (34). This supports the current study where co-incubation of exogenous adiponectin reduced the translocation of NF-ĸβ (p65) induced by oxHDL. Many other studies also have proven the ability of adiponectin to inhibit the NF-ĸβ signaling pathway. However, a limited number of studies have discussed directly the protective properties of adiponectin towards detrimental effects caused by oxHDL. However, a study showed that adiponectin has the ability to reduce the detrimental effects of oxLDL by reducing the expression of matrix metalloproteinase (MMP-9) (35). MMP-9 is known to be involved in stimulating the proliferation and migration of VSMCs into the intima layer, leading to plaque rupture (35).

Additionally, TNF-α secretion in the present study also decreased when the cells were stimulated with adiponectin. It is reported that adiponectin inhibits TNF-α stimulated calcification in HAoVSMCs through activation of AMPK (36). Besides, adiponectin is also involved in inhibiting the adhesion of monocyte to endothelial cells by inhibiting the activation of NF-kB induced by TNF-α (35). Findings in the present study strengthen the fact regarding the role of adiponectin in reducing and preventing the progression of vascular calcification and osteoblastic differentiation of VSMCs (2). Trimeric adiponectin has been proven to be involved in enhancing the differentiation of synthetic VSMC culture model which displays similar characteristics as VSMC found in intimal hyperplastic lesions. This differentiation enhances the secretion of contractile protein markers and prevents the osteoblastic transdifferentiation (37). Secretion of STAT-3 is usually parallel with the secretion of IL-6, indicating the induction of inflammatory environment. However, the results obtained in the present study need to be further analyzed since STAT-3 is not affected by oxHDL. Therefore, in the present study it indicates that the presence of trimeric adiponectin could suppress the effect of oxHDL in inducing transdifferentiation of VSMC and possibly could conserve the physiological HAoVSMCs by attenuating IL-6/WNT-5a/NF-ĸβ (p65)/osterix.

In conclusion, the present study confirms the role of oxHDL in promoting the transdifferentiation of HAoVSMCs to osteoblast-like cells by heightening the secretion of IL-6, WNT-5a, NF-ĸβ (p65), and osterix. Futhermore, the detrimental effects of oxHDL are suppressed by the presence of adiponectin. This is suggesting that adiponectin is a good potential protector and suppressor for vascular calcification. Perhaps these results could be useful for further research focussing on developing drugs for patients with the risk of having high oxHDL levels by looking at the potential protection of adiponectin against oxidation of HDL. Thus, this study will be beneficial for the development of better treatment especially in the management of obesity, chronic kidney disease, and atherosclerosis related complications in patients.
